# Efficient Identification of Transcription Factor Binding Sites with a Graph Theoretic Approach

**DOI:** 10.1155/2013/856281

**Published:** 2013-01-03

**Authors:** Jia Song, Li Xu, Hong Sun

**Affiliations:** ^1^College of Electrical Engineering, Zhejiang University, Hangzhou 310027, China; ^2^Department of Electronic and Information Technology, Suzhou Vocational University, Suzhou 215104, China

## Abstract

Identifying transcription factor binding sites with experimental methods is often expensive and time consuming. Although many computational approaches and tools have been developed for this problem, the prediction accuracy is not satisfactory. In this paper, we develop a new computational approach that can model the relationships among all short sequence segments in the promoter regions with a graph theoretic model. Based on this model, finding the locations of transcription factor binding site is reduced to computing maximum weighted cliques in a graph with weighted edges. We have implemented this approach and used it to predict the binding sites in two organisms, *Caenorhabditis elegans* and *mus musculus*. We compared the prediction accuracy with that of the Gibbs Motif Sampler. We found that the accuracy of our approach is higher than or comparable with that of the Gibbs Motif Sampler for most of tested data and can accurately identify binding sites in cases where the Gibbs Motif Sampler has difficulty to predict their locations.

## 1. Introduction

Gene regulation is one of the most important biological processes at molecular level. Recent work [1] has shown that gene regulations precisely control the expression levels of genes, which in turn controls many biological processes. In general, the regulation is performed by a binding process, where a protein called *transcription factor* binds to the promoter region of a gene. The nucleotides where the binding occurs form a *binding site*. Methods that can accurately identify the locations of binding sites in the promoter regions of genes are thus crucial for understanding the process of gene regulation.

Traditionally, TF binding sites have been characterized by a variety of different experimental approaches [2, 3]. However, predicting transcription factor binding sites with experimental approaches is often expensive and time consuming. Recently, with the large amount of sequence data, computational approaches have become an important alternative to predicting the transcription factor binding sites [4–9]. Most of the available computational approaches process the promoter regions of a set of homologous genes and recognize binding sites by identifying subsequences that are similar in sequence content. Most of these approaches employ a randomized sampling procedure to identify these subsequences and thus cannot guarantee the prediction accuracy.

For example, approaches based on Gibbs sampling randomly select a candidate subsequence of a fixed length from each sequence. A sequence is arbitrarily selected and each subsequence of the same length in the sequence is aligned to a profiling model obtained from the subsequences selected on other sequences, and each subsequence in the selected sequence is thus associated with a probability value which is the alignment score. One of the subsequences is then selected based on the distribution of the probability values of these subsequences, and a new set of subsequences is thus obtained. The procedure can be repeated until the maximum allowed number of iterations has been reached or a set of satisfying local optimal subsequences have been found [4, 5, 10]. Consensus used a greedy algorithm to align functionally related sequences and applied the algorithm to identify the binding sites for the *E. coli* CRP protein [11]. Bailey and Elkan [12, 13] used the Expectation maximization technique to fit a two-component mixture model to find binding sites and developed a software MEME+. MEME+ performed better than their previous MEME software [13]. However, its accuracy for identifying transcription factor binding sites is far from being satisfactory.

Genetic algorithms simulate the process of Darwin evolutionary process to find a local optimal solution for an optimization problem. Approaches based on GAs start with an initial population of a certain size. Each individual in the population is a valid solution for the problem. The individuals in the population then go through selection, crossover, and mutation based on certain methods and probabilities to evolve to the next generation. This evolutionary procedure keeps going until the maximum allowed number of generations is reached or the difference between the results falls into a small threshold. Genetic algorithms have been applied to predict the transcription factor binding sites such as FMGA [14]. FMGA was declared to have better performance than Gibbs Motif Sampler [5] in both accuracy and computation time. MDGA [15] is another program that used genetic algorithms to find motifs in homologous sequences. It used information content to evaluate the fitness of an individual during the evolution. MDGA achieved higher accuracy than Gibbs sampling algorithm based approaches while requiring less computation time [15].

In this paper, we develop a new approach that can predict the transcription factor binding sites without using a sampling procedure to select subsequences. Our approach uses a graph to model all subsequences in the promoter regions of the homologous genes and the similarity between any pair of subsequences that are from different promoter regions. In particular, each subsequence is represented by a vertex in the graph, and two vertices are joined by an edge if the two corresponding subsequences are from different promoter regions and their similarity is higher than a threshold. The threshold can be determined using the base compositions of the promoter regions and is guaranteed to be statistically significant. Each edge in the graph is associated with a weight value which is the similarity of the two corresponding subsequences. We then compute the maximum weighted clique in the graph, and the subsequences represented by the vertices in the clique are the transcription factor binding sites.

In order to efficiently compute the maximum weighted clique in the graph, we developed an iterative approach to preprocess the vertices in the graph and remove the vertices that cannot be in the clique from the graph, the size of the graph can thus be significantly reduced by applying this technique to it. After the preprocessing stage, the algorithm exhaustively enumerates all cliques that contain as many vertices as the number of promoter region sequences in the data set and returns the one with the largest weight value.

We have implemented this approach in a computer program with C++ programming language and used the program to predict the binding sites for two organisms, *Caenorhabditis elegans* and *mus musculus*. We evaluated the prediction accuracy of our approach based on the testing data sets and compared it with that of the Gibbs Motif Sampler [5]. Our testing results showed that our approach can achieve an average developer score of 0.90 on the test data, which is higher than or comparable with that of the Gibbs Sampler. In addition, our approach can accurately identify the binding sites in some cases where the Gibbs Motif Sampler has difficulty to locate the binding sites, which is one important advantage of our approach over the Gibbs Motif Sampler.

## 2. Method

### 2.1. Notations and Problem Description

Given a graph *G*  = (*V*, *E*), a *clique *in *G* is a vertex subset *C* such that every pair of vertices in *C* is joined by an edge in *G*. If each edge in *G* is associated with a weight value, a *maximum weighted clique* is a clique where the sum of the weight values of all edges in the clique is the maximum of all cliques in *G*. The *degree* of a vertex is the number of vertices that are adjacent to it in *G*.

Given the sequences of the promoter regions of a set of *k* homologous genes, *S*_1_, *S*_2_,…, *S*_*k*_, the goal of the problem. The goal of the problem is to select a subsequence of a given length *L* from each sequence such that the sum of the similarities between all pairs of selected sequences is maximized. To compare two subsequences and evaluate their similarity, we perform a pair wise alignment between them, and the alignment score is used as a measure of the similarity. We show later in the paper that this problem can be solved by computing the maximum weighted clique in a graph.

### 2.2. The Algorithm

**Figure 1:**
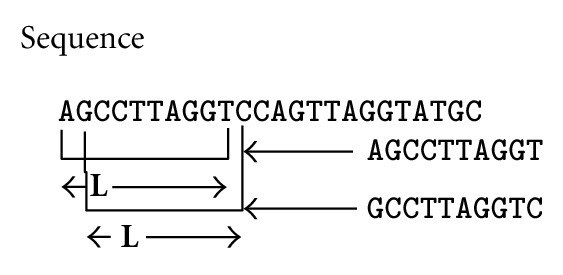
Shows the procedure used to break a sequence into its subsequences.

Given a set of DNA sequences *S*_1_, *S*_2_,…, *S*_*k*_ with similar transcription factor binding sites, we take each *S*_*i*_ where *i* ∈ [1, *K*] and divide it into *N*-*L* subsequences of length *L* as described in Figure 1, where *N* is the number of nucleotides in *S*_*i*_. In particular, we choose the subsequence that starts from each nucleotide in *S*_*i*_ and contains *L* nucleotides. It is not difficult to see that the number of such subsequences is *N*-*L*. 

We then construct a graph *G* such that each vertex of the graph represents a subsequence. Subsequences from the same sequence are placed in one *column,* and all vertices in *G* can thus be partitioned into *k* disjoint columns. A sample of randomly generated and normally distributed sequence comparison scores is used to calculate a threshold which is then used to determine which sequences are similar. The algorithm starts with the first column and selects a vertex in that column and aligns its corresponding subsequence to that of every other vertex that is from a different column. If the alignment score of two subsequences is higher than the threshold, we make their vertices adjacent in *G*. The algorithm repeats the above process for each vertex until all vertices in the graph have been processed. 

After all the edges have been added to the graph, we proceed to preprocess the graph and remove the vertices that cannot be in a clique of size *k*. In particular, we examine the degree of each vertex, and if the degree of a vertex is less than *k*, we remove it from *G*. This procedure is applied iteratively to all vertices in the graph until the size of the graph cannot be further reduced.

The algorithm then starts enumerating all *k*-cliques in the graph and computes the weight of each clique. To this end, the algorithm assigns an integer id between 1 and *k* to each column in the graph and starts with columns 1 and 2. In particular, all edges that connect a vertex from column 1 and a vertex from column 2 are included in a set *S*. *S* is maintained by the algorithm to store cliques. Initially, *S* contains a set of edges, which are in fact cliques on two vertices. The algorithm then proceeds to examine vertices in columns 3 through *k*. After the algorithm completes processing column *j*, *S* contains a set of cliques of size *j* in *G*. For every vertex *u* in column *j* + 1, the algorithm checks each *j*-clique *M* in *S* to examine whether *u* and *M* can together form a *j* + 1-clique. In other words, the algorithm examines whether *u* is adjacent to every vertex in *M* or not. If it is the case, *u* and *M* are combined into a single *j* + 1-clique and included in *S*. After every vertex in column *j* + 1 has been processed, the algorithm examines the cliques in *S* and removes all *j*-cliques from *S*. It is not difficult to see that after all *k* columns have been processed, *S* contains a set of *k*-cliques in *G*. It then computes the weight value of each clique in *S* by adding up the weight values of all edges in the clique and outputs the clique with the largest weight value.

The matrix used to generate the comparison score is a log odds ratio matrix for comparing DNA nucleotide sequences. This matrix was developed by Chiromante et al. [16]. They followed a common approach used in protein alignment and determined substitution score by using a set of trusted aligned symbol pairs and using log odds ratio [16]. The matrix used for the alignment of short subsequences is shown in the Table 1.

In order to compute the threshold of similarity value that is used to construct the edges in *G*, we first compute the base composition of all promoter region sequences. Based on the percentage of each nucleotide in the base composition, we randomly generate two subsequences, each of which contains *L* nucleotides. We then perform a pair wise alignment between the two generated subsequences. The alignment can generate an alignment score. We repeat the procedure for a sufficiently large number of times, and we thus can obtain a large collection of alignment scores. This collection of alignment scores in fact describes the distribution of alignment scores between two subsequences from the given base composition. We then choose a confidence value *p* and find the smallest value *c* such that the percentage of the alignment scores higher than *c* in the collection is not larger than *p*. The value of *c* is then used as the threshold of similarity to construct graph *G*.

### 2.3. Time Complexity

Each iterative step in the preprocessing stage of the algorithm may need up to *O*(*N* ^2^) computation time, where *N* is the number of nucleotides in each promoter region sequence. Since each iterative step removes at least one vertex from the graph. The preprocessing stage may need up to *O*(*N* ^3^) computation time. We use *W* to denote the maximum number of vertices in a column in the graph after the graph is preprocessed. The number of *k*-cliques in the graph is at most *O*(*W* ^*k*^). The computation time needed to check whether a vertex can be added into an existing clique to form a larger clique is at most *O*(*k*). The computation time needed to compute the maximum weighted clique in the preprocessed graph is thus at most *O*(*kW* ^*k*^). Putting the preprocessing stage and the clique enumeration stage together, the algorithm needs at most *O*(*N* ^3^ + *kW* ^*k*^) computation time. 

### 2.4. Experimental Results

We have implemented our approach with C++ programming language and used it to predict the transcription factor binding sites in the promoter regions of three genes that were selected from two organisms *Caenorhabditis elegans* and *mus musculus*. One data set is selected from the promoter region of a gene of *Caenorhabditis elegans,* and the remaining two are selected from the promoter regions of two genes of *mus musculus*.  Table 2 provides a description of the testing data we have used to test the accuracy of our approach.

In order to evaluate the accuracy of our approach, we compare the binding sites predicted by our approach with the correct binding sites for all sequences in the data set. A developer score is computed to provide a measure of the accuracy. The developer score is computed based on the deviation of the predicted starting position of a binding site from its real starting position. In particular, for a binding site of length *L*, if the deviation is *D*, the developer score *d* of the prediction result is then computed as follows:

(1) $$\begin{array}{c} d=\max\left\{1-\frac{D}{L},0\right\}. \end{array}$$

It is not difficult to see that the developer score is a measure of the accuracy of a predicted binding site. If the predicted binding site is completely correct, the deviation *D* is 0, and the developer score is thus 1.0. On the other hand, if the predicted binding site is completely incorrect, it does not even intersect the real binding site, the deviation *D* is at least *L*, and the developer score is thus 0.0. In general, the developer score of a predicted binding site is a positive real number between 0.0 and 1.0. A higher value of the developer score indicates a more accurate prediction result.

We also used the Gibbs Motif Sampler [5] to predict the binding sites on the same data sets and evaluate the accuracy of its prediction results with the developer score. We compare the accuracy of our approach with that of the Gibbs Motif Sampler. Tables 3, 4, and 5 provide the accuracy of our approach and that of the Gibbs Motif Sampler on three tested data sets in terms of the developer score.

It is evident from the testing results that our program can achieve an average developer score of 0.90 for the testing data sets. This indicates that our approach can identify the binding sites with accuracy comparable with or better than that of the Gibbs Motif Sampler for most of the tested sequences. In addition, in some cases where the Gibbs Motif Sampler has difficulty to locate the binding sites, our program can identify the binding sites with high accuracy. For example, for sequences 2, 3, and 18 in the data set for *mus musculus* that contains binding sites of length 11, the Gibbs Motif Sampler fails to identify the correct locations of binding sites while our approach identifies the binding sites for all sequences with high accuracy. The Gibbs Motif Sampler significantly outperforms our approach only in sequence 16 in the data set for *Caenorhabditis elegans*, where our approach fails to identify the correct location of binding site while the Gibbs Motif Sampler identifies its correct location without any error. It is worth mentioning that our approach achieves significantly higher prediction accuracy for all sequences in the data set for *mus musculus* that contains binding sites of length 11.

## 3. Conclusions

In this paper, we developed a novel approach that can efficiently and accurately predict the transcription binding sites in the promoter regions of genes. Our approach uses a graph model to describe the subsequences in the promoter regions of homologous genes and their relationships. The problem is then reduced to a graph optimization problem. In order to efficiently compute the optimal solution of the problem, we developed a preprocessing technique that can significantly reduce the size of the graph. Our testing results on the sequence data from two organisms *Caenorhabditis elegans* and *mus musculus* showed that our approach can achieve prediction accuracy higher than or comparable with that of the Gibbs Motif Sampler. 

We believe the performance of our approach can be further improved if we employ a weighted scoring scheme that can assign different relative weight values to the pair wise matching scores obtained on different positions in the subsequences. It is well known that mutation is much more likely to occur in nucleotides near the boundary of the binding sites than those near the center of the binding sites. Lower values of relative weights thus should be assigned to the matching scores obtained on nucleotides near the boundary of the binding sites. Determining the relative weights that can maximize the accuracy of prediction is an interesting problem and would be a part of our future work.

## Figures and Tables

**Table 1:** Shows the matrix used to compute comparison scores.

	A	C	G	T
A	100	−123	−28	−109
C	−123	91	−140	−28
G	−28	−140	91	−123
T	−109	−28	−123	100

**Table 2:** Information on the data sets we have used to test the accuracy of our approach.

Data set number	Number of sequences	Length of each sequence	Length of the binding site	Organism the data set describes
1	27	11-12	6	*Caenorhabditis elegans *
2	20	11–13	6	*mus musculus *
3	20	19–23	11	*mus musculus *

**Table 3:** The developer score of the predicted binding sites in data set 1 for both our approach and the Gibbs Motif Sampler. The actual binding sites are marked by nucleotides in capital letters.

Actual binding site	Binding site predicted by our approach	Binding site predicted by Gibbs Motif Sampler	Developer score of our approach	Developer score of Gibbs Motif Sampler
GAAACCctgtta	AAACCC	GAAACC	0.83	**1.00**
GAAGCCcttcaa	AAGCCC	GAAGCC	0.83	**1.00**
GAAGCCgcaaaa	GAAGCC	GAAGCC	1.00	1.00
GAAGCCcctcac	GAAGCC	GAAGCC	1.00	1.00
GAAGCCaattat	GAAGCC	GAAGCC	1.00	1.00
GAAGCCttagaa	GAAGCC	GAAGCC	1.00	1.00
GAATCCttagat	GAATCC	GAATCC	1.00	1.00
GAAACCttgcaa	GAAACC	GAAACC	1.00	1.00
GAAGCCaatcat	GAAGCC	GAAGCC	1.00	1.00
GAAACCttatga	GAAACC	GAAACC	1.00	1.00
GAAACCtttcaa	GAAACC	GAAACC	1.00	1.00
GAAACCatagac	GAAACC	GAAACC	1.00	1.00
GAAGCCttatta	GAAGCC	GAAGCC	1.00	1.00
GAAGCCcccaaa	GAAGCC	GAAGCC	1.00	1.00
GAAGCCgtagct	GAAGCC	GAAGCC	1.00	1.00
GAAGCCacaatt	GAAGCC	GAAGCC	1.00	1.00
GAAGCCgtgttt	GAAGCC	GAAGCC	1.00	1.00
GAAACCttatct	GAAACC	GAAACC	1.00	1.00
GAAGCCgtacaa	GAAGCC	GAAGCC	1.00	1.00
GAAGCCtacaaa	GAAGCC	GAAGCC	1.00	1.00
GAAACCttattt	GAAACC	GAAACC	1.00	1.00
GAAGCCgtaaaa	GAAGCC	GAAGCC	1.00	1.00
GAAGCAccttat	AAGCAC	GAAGCA	0.83	**1.00**
GAAGCCttaaaa	GAAGCC	GAAGCC	1.00	1.00
GAAGCCgtagat	GAAGCC	GAAGCC	1.00	1.00
GAAGCCactttt	GAAGCC	GAAGCC	1.00	1.00
GAATCCctacaa	GAATCC	GAATCC	1.00	1.00

**Table 4:** The developer score of the predicted binding sites in data set 2 for both our approach and the Gibbs Motif Sampler. The actual binding sites are marked by nucleotides in capital letters.

Actual binding site	Binding site predicted by our approach	Binding site predicted by Gibbs Motif Sampler	Developer score of our approach	Developer score of Gibbs Motif Sampler
tgaatacCACGTG	CACGTG	CACGTG	1.00	1.00
gggatCACGTGgt	CACGTG	CACGTG	1.00	1.00
attgtgCACGTGg	CACGTG	CACGTG	1.00	1.00
CACGTGggaggtac	CACGTG	CACGTG	1.00	1.00
gggtCACGTGttc	CACGTG	CACGTG	1.00	1.00
taagCACGTGgtc	CACGTG	CACGTG	1.00	1.00
CACGTGccgcgcgc	CACGTG	CACGTG	1.00	1.00
aggtataCACGTG	TACACG	CACGTG	0.67	**1.00**
AACGTGcacatcgtcc	AACGTG	CACGTT	**1.00**	0.00
AACGTGacttcgtacc	AACGTG	CACGTT	**1.00**	0.00
CACGTGatgtcctc	CACGTG	CACGTG	1.00	1.00
CACGTGaagttgtc	CACGTG	CACGTG	1.00	1.00
AACGTGacagccctcc	AACGTG	CACGTT	1.00	1.00
agtCACGTGttcc	CACGTG	CACGTG	1.00	1.00
taaatgcCACGTG	CACGTG	CACGTG	1.00	1.00
tgaCACGTGtccg	CACGTG	CACGTG	1.00	1.00
AACGTGcgtgatgtcc	AACGTG	CACGTT	1.00	0.00
catgtCACGTGcc	CATGTC	CACGTG	0.17	**1.00**
aggaatCGCGTGc	CGCGTG	Not Found	**1.00**	0.00
agttcgCACGTGc	CGCACG	CACGTG	0.67	**1.00**

**Table 5:** The developer score of the predicted binding sites in data set 3 for both our approach and the Gibbs Motif Sampler. The actual binding sites are marked by nucleotides in capital letters.

Actual binding site	Binding site predicted by our approach	Binding site predicted by gibbs motif sampler	Developer score of our approach	Developer score of gibbs motif sampler
gcacATAGGTGTAAAatggccgttgg	CATAGGTGTAA	CACATAGGTGTAA	**0.91**	0.73
ctcgcacCCAGGTGTGAAgttctggt	CCCAGGTGTGA	CACCTGGGTGCGA	**0.91**	0.00
acGTAGGTGCGAAtctatcttagtgc	CGTAGGTGCGA	Not Found	**0.91**	0.00
gcgagatgtaacatGTAGGTGTGAAa	TGTAGGTGTGA	CATGTAGGTGTGA	**0.91**	0.73
ctttactcacCTAGGTGTGAAtgaag	CCTAGGTGTGA	CACCTAGGTGTGA	**0.91**	0.73
gcacGTAGGTGCTACttttttgtaa	CGTAGGTGCTA	CACGTAGGTGCTA	**0.91**	0.73
acatagtgacacCTAGGTGTGAAatt	CCTAGGTGTGA	CACCTAGGTGTCA	**0.91**	0.73
cgtcacgcGTAGGTGTTACaatgtgg	CGTAGGTGTTA	CGCGTAGGTGTTA	**0.91**	0.00
gtcatGTAGGTGTGAAtatagcgccc	TGTAGGTGTGA	CATGTAGGTGTGA	**0.91**	0.73
tttgacacCTAGGTGTCATattccac	CCTAGGTGTCA	CACCTAGGTGTCA	**0.91**	0.73
tatcgcacCTAGGTGTGACaatcatc	CCTAGGTGTGA	CACCTAGGTGTGA	**0.91**	0.73
gcaaGTAGGTGTGAAatctcaacgga	AGTAGGTGTGA	CAAGTAGGTGTGA	**0.91**	0.73
acatagtgacacCTAGGTGTGAAattc	CCTAGGTGTGA	CACCTAGGTGTCA	**0.91**	0.73
gtggaatatgacacCTAGGTGTCAAa	CCTAGGTGTCA	CACCTAGGTGTCA	**0.91**	0.73
acacCTAGGTGTGAAattcagatata	CCTAGGTGTGA	CACCTAGGTGTGA	**0.91**	0.73
attagtcacacCTAGGTGTGAAgagc	CCTAGGTGTGA	CACCTAGGTGTGA	**0.91**	0.73
ccagtatcacacTTAGGTGTTACatc	CTTAGGTGTTA	CACTTAGGTGTTA	**0.91**	0.73
tctactaacagGTAGGTGTTACttgt	GGTAGGTGTTA	CAGGTAGGTGTTA	**0.91**	0.73
gcggAAAGGTGTGAAatcacaccatt	GAAAGGTGTGA	Not Found	**0.91**	0.00
gaattcacacTTAGGTGTGAAat	CTTAGGTGTGA	CACTTAGGTGTGA	**0.91**	0.73
